# Programmable Acoustic Holography using Medium‐Sound‐Speed Modulation

**DOI:** 10.1002/advs.202301489

**Published:** 2023-06-07

**Authors:** Mingxin Xu, Jizhen Wang, William S. Harley, Peter V. S. Lee, David J. Collins

**Affiliations:** ^1^ Department of Biomedical Engineering University of Melbourne Melbourne Victoria 3010 Australia; ^2^ Graeme Clarke Institute University of Melbourne Parkville Victoria 3052 Australia

**Keywords:** acoustic hologram, fluid identification, micromanipulation, programmable acoustic field

## Abstract

Acoustic holography offers the ability to generate designed acoustic fields to manipulate microscale objects. However, the static nature or large aperture sizes of 3D printed acoustic holographic phase plates limits the ability to rapidly alter generated fields. In this work， a programmable acoustic holography approach is demonstrated by which multiple discrete or continuously variable acoustic targets can be created. Here, the holographic phase plate encodes multiple images, where the desired field is produced by modifying the sound speed of an intervening fluid media. Its flexibility is demonstrated in generating various acoustic patterns, including continuous line segments, discrete letters and numbers, using this method as a sound speed indicator and fluid identification tool. This programmable acoustic holography approach has the advantages of generating reconfigurable and designed acoustic fields, with broad potential in microfluidics, cell/tissue engineering, real‐time sensing, and medical ultrasound.

## Graphical abstract

A programmable acoustic holography approach is demonstrated, where multiple discrete or continuously variable acoustic patterns can be generated. This approach encodes multiple images on a holographic phase plate, where the desired field is generated by modifying the sound velocity of the intervening fluid medium. This acoustic holographic approach has broad potential in microfluidics, cell/tissue engineering, real‐time sensing, and medical ultrasound.

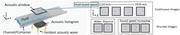

## 1 Introduction

Acoustic holography generates designed acoustic fields by modulating the interference of wavefronts in free space,^[^ ^1^, ^2^, ^3^, ^4^, ^5^, ^6^, ^7^, ^8^ ^]^ with emerging applications in cell/tissue engineering,^[^ ^9^, ^10^, ^11^ ^]^ medical ultrasound,^[^ ^12^, ^13^, ^14^, ^15^ ^]^ particle manipulation,^[^ ^16^, ^17^, ^18^, ^19^ ^]^ and particle detection,^[^ ^20^ ^]^ where acoustic fields can generate rapid microparticle and cell motion via the emergence of time‐averaged pressure and velocity fields.^[^ ^21^, ^22^ ^]^ Acoustic holography is achieved by modifying the phase and/or amplitude of the acoustic waves,^[^ ^23^, ^24^, ^25^, ^26^ ^]^ where back‐calculated spatial phase/amplitude maps typically generate a single designed acoustic “image” at a select distance from the modulation plane.^[^ ^27^, ^28^, ^29^ ^]^ Compared to other acoustic manipulation approaches, acoustic holograms can readily produce unique particle patterns beyond the nodal lines and grids that are otherwise generated in acoustofluidic devices.^[^ ^30^, ^31^, ^32^ ^]^ Additionally, while select acoustic approaches can generate non‐periodic patterns,^[^ ^33^, ^34^, ^35^, ^36^, ^37^ ^]^ acoustic holograms have the advantage of decoupling structures that modify the acoustic field from the region they are utilized, making them more widely applicable beyond microfluidic devices. A limitation of most static metasurface‐based holograms^[^ ^29^, ^38^, ^39^ ^]^ is that only a single and static acoustic field can be generated at a target plane for a given hologram, limiting the range of activities that can be performed, whereas applications in tissue engineering and 3D printing benefit from the incorporation of designed heterogeneity.^[^ ^40^, ^41^ ^]^

Flexible and reconfigurable acoustic fields can be generated using phased array transducers (PATs),^[^ ^17^, ^42^, ^43^, ^44^, ^45^, ^46^ ^]^ in which many ultrasonic devices are individually actuated with varying phase/amplitude. However, the aperture size and drive circuitry of PATs add cost and complexity as the element number increases,^[^ ^47^, ^48^ ^]^ limiting the spatial resolution at which PATs can create sophisticated wavefront manipulation.^[^ ^49^, ^50^ ^]^ This also limits the application of PATs to acoustic holography, especially for complex patterns and high‐frequency actuation, which would require tens of thousands of microscale and individually addressable transducers to generate high‐resolution acoustic fields at scales and frequencies comparable to static (typically printed) metasurface plates.^[^ ^42^, ^51^, ^52^, ^53^, ^54^ ^]^

To remedy this, improvements in programmable acoustic holograms have been demonstrated with the ability to modulate the acoustic field produced at a given plane,^[^ ^55^, ^56^, ^57^ ^]^ where, for instance, a reconfigurable hologram with 10000 addressable pixels utilized a single transducer combined with electrolytically produced air bubbles from a complementary metal‐oxide semiconductor (CMOS) array.^[^ ^56^ ^]^ In that work, patterned air bubbles introduced acoustic impedance mismatches, resulting in spatially selective binary acoustic transmission. However, such binary‐amplitude‐based holography yields a lower information density compared to phase‐encoded holograms,^[^ ^56^, ^58^ ^]^ and the implementation of binary‐amplitude‐based programmable holography is limited by the complex multilevel fabrication/control required and the time required to regenerate bubble layers. An alternative programmable holography approach utilizes stackable holograms,^[^ ^57^ ^]^ whereby a single acoustic phase plate encodes multiple images that manifest at different, spatially separated target planes. However, creating different pressure fields at a given location requires mechanically shifting the hologram's position, increasing complexity and limiting application to integrated systems.^[^ ^59^ ^]^ Additionally, manually reconfigurable acoustic modulators have been demonstrated,^[^ ^55^, ^60^, ^61^, ^62^, ^63^, ^64^ ^]^ although these modulators generally have large apertures, large element dimensions and limited pixel numbers, limiting their application to high‐resolution acoustic fields. Further, the generation of holographic images by frequency multiplexing has been demonstrated numerically,^[^ ^65^ ^]^ however the requirement of frequency diversity of acoustic sources and discrete image transformations limit its application.

To create multiple programmable particle patterns in the same device, here we introduce an acoustic hologram approach based on modifying the sound velocity of a fluid medium. This acoustic hologram generates different acoustic patterns at a single target plane by changing the fluid medium through which acoustic wavefronts propagate. The transfer functions of the propagating wave are defined by the sound velocities of the medium and are encoded in a hologram with more than 40000 phase pixels. We verify the ability of the programmable hologram to generate various acoustic patterns including migrating continuous line segments, letters and numbers, respectively. Furthermore, the programmable holography can serve as a measurement tool for fluid media, effectively serving as a continuous sound speed indicator. Whereas prior work requires the use of complex microelectronic arrays or electromechanical systems to generate programmed acoustic fields at a given target plane, this approach utilizes simple fabrication/control methods with fast modulation of the target acoustic field, which lends itself to a diverse range of applications in microfluidics devices,^[^ ^66^ ^]^ tissue engineering,^[^ ^67^ ^]^ real‐time sensing,^[^ ^68^ ^]^ and medical ultrasound.^[^ ^6^ ^]^

## 2 Methods

**Figure 1:**
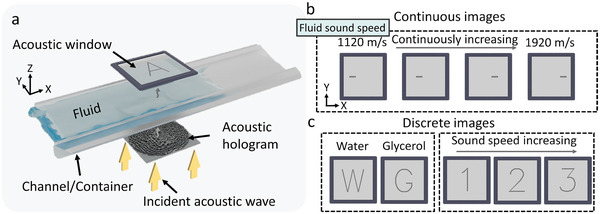
Schematic diagram of sound‐speed programmable holography. a) Incident acoustic waves are modulated by an acoustic hologram, generating different target acoustic fields in the acoustic window according to the sound velocity of the fluid. Particles in the acoustic window are patterned by the acoustic field. Programmable holograms can generate b) continuously variable and c) discrete images.

**Figure** **1**a shows a schematic diagram of programmable holography based on sound velocity modulation. Incident sound waves are modulated by a 3D‐printed acoustic hologram phase plate and travel through the fluid media into the acoustic window. Multiple target acoustic fields are encoded in the hologram. The acoustic field that manifests in the acoustic window is determined by the fluid sound velocity of the intervening media. Polydimethylsiloxane (PDMS) particles are used to visualize the acoustic field. The holograms can be programmed to display multiple images continuously (Figure 1b) and discretely (Figure 1c), respectively, as the sound speed of the medium changes. Figure 1b shows that the position of the line segments shifts as the fluid sound velocity increases, permitting the continuous measurement of sound speed within a target sound speed range. Figure 1c shows that multiple discrete images can be encoded in a single hologram. Holograms encoded with specific sound velocities can generate adjustable acoustic fields for fluid identification (left). These acoustic fields can serve as a simple visual indicator of a vessel's contents or to display programmed images at specific sound velocities (right).

**Figure 2:**
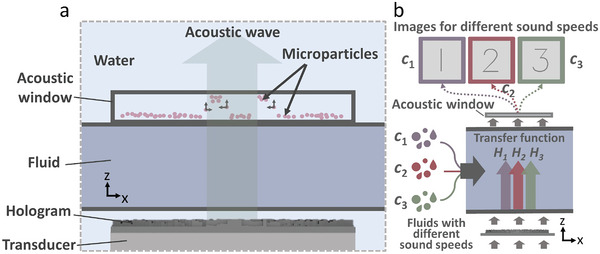
Principles of sound‐speed programmable holography. a) The acoustic waves generated by the transducer are modulated by the hologram and travel through a changeable fluid into the acoustic window, which patterns microparticles by concentrating them at the maximum acoustic intensity locations. b) Images corresponding to different medium sound speeds (and transfer functions) are encoded in a hologram. For the same incident wave, different images can be generated on the plane at z according to the medium sound speed. Supporting Information Figure S1 (Supporting Information) shows the dimensions and photos of the device.

**Figure** **2**a shows the principles of wave propagation and the visualization of the pressure field at the target plane. Acoustic waves generated by piezoceramic transducers (see Experimental Section for experimental setup) are modulated by the hologram and pass through a coupling layer (water) into the fluid channel and acoustic window. The acoustic waves modulated by the hologram pass through the fluid into the acoustic window, where the microparticles are pushed up and concentrated at the top of the acoustic window (along +*z*).^[^ ^56^, ^69^ ^]^ The colored PDMS microparticles visualize the target pressure fields. The microparticles in the image plane can only be moved laterally by the acoustic radiation force as the top film constrains the movement of the particle in the +*z* direction, where the PDMS microparticles have a negative acoustic contrast and therefore move along acoustic force potential gradients towards regions with the highest acoustic field intensity.^[^ ^70^, ^71^ ^]^

To design the sound‐speed programmable holograms, we use a modified iterative angular spectrum approach (IASA)^[^ ^72^, ^73^, ^74^ ^]^ to obtain the phase distribution of the hologram plane that results in a desired image in the target plane (see Experimental Section for details). Briefly, the angular spectrum of the pressure field at the hologram plane is multiplied by a transfer function to obtain the angular spectrum (and therefore the pressure field) of the target image plane at *z*. The transfer function *H* is expressed as:

(1) $$H\;\left(k_{m},z\right)=e^{jz\sqrt{k_{m}-\alpha }}$$

(2) $$\alpha =k_{x}^{2}+k_{y}^{2}$$

where *z* is the distance between the image plane and the hologram plane, *k*_m_ = 2*πf*/*c*_m_ is the wave number, *c*_m_ is the medium sound speed, and *k*_x_ and *k*_y_ are the Fourier frequencies in *x* and *y* directions, respectively, where *k*_x_, *k*_y_, and *α* are constants. Equation 1 describes that different medium sound velocities *c*_m_ (and *k*_m_) correspond to different transfer functions encoded in a hologram, which correspond to different images generated in the plane at *z*, as shown in Figure 2b.

## 3 Experimental Section

**Figure 3:**
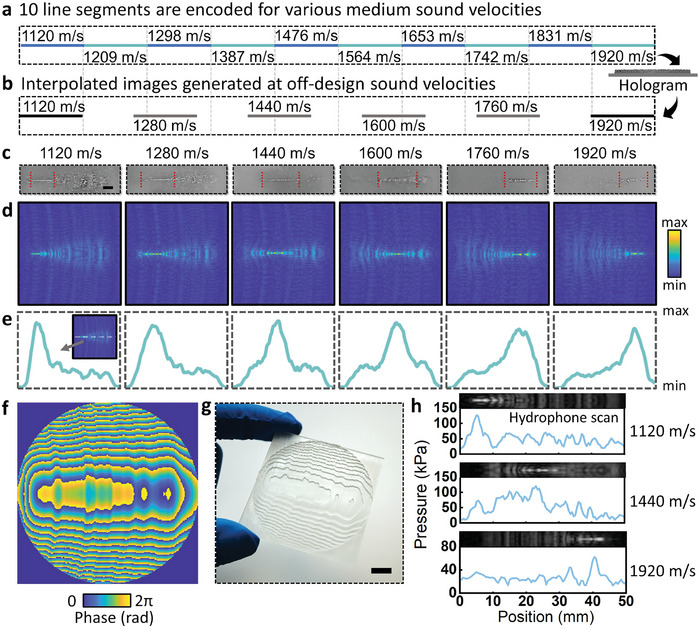
Generating interpolated images at off‐design sound velocities. a) Encoded holographic target line segment images (ten), with increasing sound velocity from left to right (from 1120 to 1920 m s^−1^). b) Encoded holograms can generate interpolated images at arbitrary sound velocities in the range. c) Experimental results using PDMS microparticles at the sound velocities from b). d) Corresponding simulation results and e) midline acoustic pressure distributions. f) Acoustic hologram phase distribution. g) Printed hologram. Hydrophone scanning results and corresponding simulation results for medium sound velocities of h) 1120, 1440, and 1920 m s^−1^. Scale bars are 5 mm.

Whereas multiple discrete images had been demonstrated previously (although at different target plane locations),^[^ ^27^, ^57^ ^]^ here interpolated continuous images can be generated by encoding interlinked images in multiple holograms at different sound velocities within a given sound speed range. **Figure** **3**a shows ten decile segments in the sound speed range of 1120 m s^−1^ to 1920 m s^−1^, arranged end to end from left to right. The line segment images were encoded in a hologram according to their corresponding transfer functions (by Equation 1). These line segments were discretely coded for specific sound velocities, whereas the displayed images were continuously interpolated at arbitrary sound velocities, as shown in Figure 3b. By using a set of holograms that were continuously arranged end‐on‐end, the interpolated images were the result of the transfer function interpolating between neighboring designed sound velocities.

Figure 3c,d show the experimental and simulation results corresponding to the mediums with the sound speeds given in Figure 3b, where the operating frequency was 2.26 MHz. Each plot in Figure 3e was the simulation result for the acoustic pressure of the midline in Figure 3d, showing a maximum intensity line location that shifts to the right with increasing sound speed. Importantly, the experimental sound speeds assessed do not need to exactly correspond to any of the encoded sound speed values, where the quality of the focussing was equivalent regardless of whether the utilized sound speed was close to or between the encoded ones, making this appropriate as a visual indicator for continuous sound speed sensing. This approach was conceptually simple, robust, and does not require complex peripherals such as display/signal processing equipment, making it potentially suitable for integration with applications including microfluidics, microelectromechanical systems, and wearable systems.

The phase distribution of the circle‐shaped hologram was shown in Figure 3f. The 3D printed hologram (see Experimental Section for details) with 50 mm × 50 mm dimensions was shown in Figure 3g, which contains 208 × 208 elements where each pixel has a length/width of 240 µm. Beyond the formation of particle patterns in the designed configuration, hydrophone scans were performed to demonstrate the shifting maxima locations with changing sound speed, with the hydrophone scanning results for different c_m_ and their simulated acoustic pressure field shown in Figure 3h.

**Figure 4:**
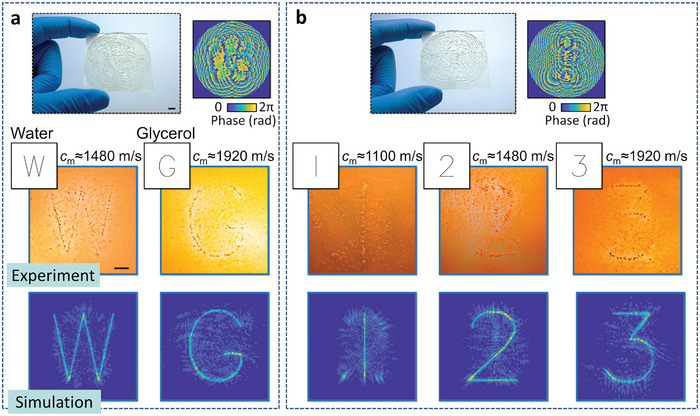
Multiple images encoded in static acoustic holograms. a) Here a hologram coded according to the initials of the media (water, left, and glycerol, right) provides an intuitive visual representation of the media. b) Number‐encoded hologram. Different numbers are generated with the use of different media (methanol, water and glycerol, from left to right). Scale bars are 5 mm.

Multiple discrete, unrelated images can further be generated at the same target plane by altering the fluid medium, where **Figure** **4** shows holograms programmed with letters and numbers. The printed holograms and phase distribution were shown at the top of each figure, with experimental and simulation results below. The letter‐encoded hologram in Figure 4a, for instance, generates the initials of the media according to their corresponding sound velocities. Here the letters “W” and “G”, were generated by utilizing water (*c*_m_≈1480 m s^−1^) and glycerol (*c*_m_≈1920 m s^−1^), respectively. The number‐encoded hologram in Figure 4b contains phase information with numbers from 1 to 3, which intersect the target plane at the designed media sound speeds (1100, 1480, and 1920 m s^−1^, respectively). Supporting Information Movie S1 (Supporting Information) shows the transition between the different hologram images in Figure 4b, in which the medium was changed from glycerol to water and then methanol (numbers 3→1), demonstrating the ability to change the generated particle pattern without the need to replace the hologram plate or shift its position.

**Figure 5:**
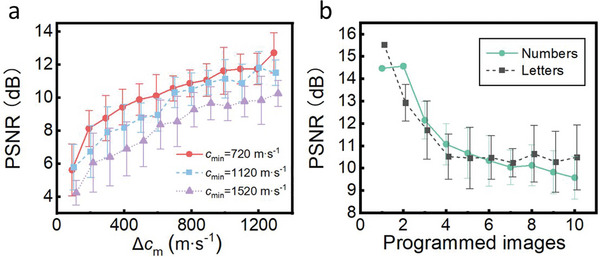
Effect of design parameters on image quality, indicated by the peak signal‐to‐noise ratio (PSNR). a) Increased sound velocity range (Δ*c*_m_) and reduced minimum sound velocity (*c*_min_) improve the reconstructed image quality. b) Image quality degrades as the number of images programmed in a hologram increases. PSNR is calculated based on the normalized target image magnitude and binary input image windowed to 108 × 68 pixels. Figure S4 (Supporting Information) shows the resulting images for Figure 5b.

Whereas a handful of images were generated in Figure 4 for a given printed hologram plate, there was a tradeoff between image quality and the number of images contained in a hologram, as well as the separation between the distances at which hologram images were manifested. The effect of design parameters on the reconstruction quality of the holographic acoustic field was examined in Figure 5, including sound speed differential and number of images. The peak signal‐to‐noise ratio (PSNR) was used to describe the quality of the reconstructed images, where a higher PSNR generally indicates the better reconstruction quality.^[^ ^75^, ^76^ ^]^ **Figure** **5**a shows the effect of the sound velocity differential (Δ*c*_m_) used for encoding on the reconstruction quality for the design in Figure 3, where Δ*c*_m_ = *c*_max_‐*c*_min_, *c*_max_, and *c*_min_ were the maximum and minimum sound speeds within the encoded sound velocity range, respectively. The different curves represent the minimum sound velocities in the encoded sound speed range. These results show that increased Δ*c*_m_ and reduced *c*_min_ (the latter indicating a greater proportional sound speed differential for a given Δ*c*_m_) improve the quality of reconstructed images.

Figure 5b further shows the relationship between the number of images programmed in a hologram and the reconstruction quality, for a hologram of 208 × 208 pixels and each pixel width of 240 µm (Δ*c*_m_ = 800 m s^−1^ and *c*_min_≈1120 m s^−1^). The curves here represent the results for letters (A to J) and numbers (0 to 9) respectively, showing that, while there was not necessarily a strict cut‐off in the total encoded image number, the quality of reconstructed images degrades with more than a handful of letters/numbers. Here a given hologram print contains a fixed amount of information, and whereas a single‐image hologram creates constructive interference at only one plane, requiring this interference across multiple simultaneous images results in off‐target wavefront coalescence (for instance, aspects of “2” were faintly represented in the “1” image in Figure 4b). While discrete images can thus be encoded in a practically limited number, Figure 3 demonstrates that the complexity of the image also plays a significant role, with the ten images there (single joined lines) being readily encoded without degradation in image quality, resulting in an effectively continuous and horizontally shifting line that self‐interpolates between design sound velocities. Supporting Information Figure S2 (Supporting Information) shows the numbers and letters used and their corresponding PSNR, respectively. Figure S3 (Supporting Information) shows the effect of media with different power law absorption coefficients on imaging quality, where an increased attenuation coefficient within a range result in a higher PSNR of the image. Figure S4 (Supporting Information) shows the resulting images for Figure 5b.

### 3.1 Design of Acoustic Holograms

A modified iterative angular spectroscopy method (IASA) was used to calculate the phase distribution of the programmable hologram. The calculation steps were as follows. 1)Transform the complex pressure fields of the image planes into their respective angular spectra through Fourier transform; 2) propagate the angular spectra along the −*z* axis to the hologram plane based on different propagation functions to obtain their respective angular spectra of the image plane; 3) transform the angular spectra of the holographic planes into complex pressure fields by inverse Fourier transform respectively and sum them to obtain the phase distribution of the holographic plane; 4) reset the amplitude of the holographic plane (preserve the phase); 5) propagate the angular spectrum to the image plane along the +*z* axis with their corresponding propagation functions; 6) Transform the angular spectrum of each image plane into complex pressure field and reset the amplitudes to the designed images.

Steps (1) to (5) were repeated until the image plane quality converges. The hologram phase distribution obtained in (3) was used to generate the 3D graphics file of the hologram, in which the height of each pixel, *H*_p,_ is expressed as

(3) $$H_{p}=\frac{\Delta \varphi }{\left(k_{m}-k_{0}\right)}+H_{0}$$

where Δ*φ* is the phase shift of the pixel, *k*_m_ and *k*_0_ are the wave number in the medium and hologram, respectively. *H*_0_ is the initial thickness of the hologram substrate, where the increasing pixel height from *H*_0_ results in an increased phase shift. For the hologram material and transducer frequency used here, a 2*π* phase shift corresponds to an *H_p_* of 3 mm (*H_0_* = 1.3 mm).

### 3.2 Fabrication of Hologram and Experiment Setup

The hologram files were printed using 3D printer (Photon Ultra, Anycubic, Guangdong, China) and UV‐sensitive resin (UV Sensitive Resin Basic, Anycubic, Guangdong, China). Each hologram consists of 208 × 208 pixels, the pixel dimensions were 240 µm × 240 µm. The washed and cured (Wash and Cure Machine, Anycubic, Guangdong, China) holograms were then placed on the surface of a ceramic transducer (H4P502000, Huajingda Electronic, Guangdong, China) with a resonance frequency of 2.26 MHz. To eliminate the formation of cavities resulting from the microstructure of the hologram, isopropanol was sprayed onto the surface of the hologram before immersing in water to enhance the hydrophilicity and prevent bubble formation. The ceramic transducer was wired to a power amplifier (TBMDA4B, Tekbox, Ho Chi Minh, Vietnam) and function generator (AFG 31252, Tektronix, OR, USA) and driven by a sine wave amplified to ≈5 W. The hollow fluid channels and acoustic windows were printed (using the same 3D printer above) and sealed with polyethylene terephthalate (PET) film at both sides to minimize acoustic impedance mismatch, in which the suspension containing PDMS microparticles was encapsulated in the acoustic window. Fluids were injected into the channel through the syringes and silicon tubes. All particles were placed in water, as shown in Figure 2a. Between taking each static image, the channel was completely flushed with fluids at the target sound velocity. Photos and videos have undergone basic adjustments (brightness, contrast, sharpness). The effect of channel height on image quality was shown in Supporting Information Figure S5 (Supporting Information), where lower heights generally result in better images. Using a smaller channel height to produce the same set of images, however, produces a thinner vertical distance over which a given image manifests in a finite‐thickness acoustic window. For practical considerations, an intermediate channel height as used here was preferred.

### 3.3 Fabrication of Colored PDMS Suspensions

PDMS (Sylgard 184, Dow Corning, MI, USA) with 10 to 1 mixing ratio was mixed with ≈2 wt.% silicone pigment (Orange Silicone Pigment, Barnes, NSW, Australia), and then mixed with solution of 1 wt.% surfactant (Pluronic F‐127, Sigma‐ Aldrich, MO, USA). The suspension was homogenized at 16 000 rpm for 20 mins in total at room temperature and then stirred at 40 °C for 4 h, followed by curing at room temperature for at least 24 h. PDMS microparticles with diameters < 30 µm were obtained by pipetting the supernatant suspension after precipitation for 10 mins (see Supporting Information Figure S6 (Supporting Information) for microscope image). See Supporting Information for details on the minimum/maximum PDMS particle sizes that can be manipulated.

### 3.4 Preparation of Fluids with Various Sound Velocities

The fluids were mixed in different proportions to obtain fluids varying in sound velocities (*c*_m_) ranging from 1120 m s^−1^ to 1920 m s^−1^. Deionized water (*c*_m_≈1480 m s^−1^) and methanol (*c*_m_≈1120 m s^−1^) were mixed in different ratios to obtain fluids with *c*_m_ < 1480 m s^−1^ and deionized water was used for fluids with *c*_m_≈1480 m s^−1^. Deionized water and glycerol methanol (*c*_m_≈1920 m s^−1^) were mixed in different proportions to obtain fluids with *c*_m_ > 1480 m s^−1^. A sound velocity gauge (WT100A, Wintact, Shenzhen, China) was used to verify the sound velocity.

## 4 Conclusion

In this work, we implement a unique approach for programmable acoustic holography and realize the generation of multiple holographic images from a single printed hologram device. This is achieved by encoding multiple images in a hologram, which generates different acoustic fields at the target plane according to the sound velocity of the utilized fluid media. Changing the fluid media results in different particle patterns being generated. By separating the target area from the changing fluid media, different hologram images can be produced without altering the water media in which the particles, here used to visualize the acoustic field, are suspended. Beyond generating arbitrary patterns, this programmable holography approach can conversely be used for continuous sound speed measurement sensing and medium identification. Compared to amplitude‐only reconfigurable holograms,^[^ ^56^ ^]^ such a phase‐based holography has the benefit of higher information density and achieves image switching without complex fabrication processes and ancillary components. We utilize this programmable sound‐speed‐based method to demonstrate the ability to generate multiple letters, numbers, and continuous line segments at a target plane, using printed holograms with ∼40000 pixels. This elegant programmable approach thus has relevant applications across domains in which arbitrary acoustic fields and/or micropatterning is desirable, including acoustofluidics, tissue engineering, real‐time sensing, and medical ultrasound.

## Conflict of Interest

The authors declare no conflict of interest.

## Supporting information

Supporting Information

Supplemental Movie 1

## Data Availability

The data that support the findings of this study are available from the corresponding author upon reasonable request.
