# Supplementary material for: Programmable Acoustic Holography using Medium‐Sound‐Speed Modulation
Source: Adv Sci (Weinh). 2023 Jun 7;10(23):2301489. doi: 10.1002/advs.202301489 (PMC10427405; doi:10.1002/advs.202301489)
Supplement: Supplementary file 1 — Supporting Information [file ADVS-10-2301489-s001.pdf]

ADVANCED SCIENCE
Open Access

Supporting Information

for *Adv. Sci.*, DOI 10.1002/advs.202301489

Programmable Acoustic Holography using Medium-Sound-Speed Modulation

*Mingxin Xu, Jizhen Wang, William S. Harley, Peter V. S. Lee and David J. Collins**

Supporting Information

Programmable acoustic holography using medium-sound-speed modulation

Mingxin Xu [a], Jizhen Wang [a], William S. Harley [a], Peter V. S. Lee [a,b] & David J. Collins [a,b,*]

[a] Department of Biomedical Engineering, University of Melbourne, Melbourne, VIC, 3010 Australia

[b] The Graeme Clark Institute, The University of Melbourne, Parkville, VIC, 3052 Australia

***** Corresponding author

E-mail: david.collins@unimelb.edu.au

Minimum and Maximum Manipulable Particle Sizes

The acoustic radiation force is dominant for particles larger than the critical particle size (a_c), while the streaming force is dominant for particles smaller than a_c. The critical particle size, a_c, is written as [1]:

$$a_c \sim \sqrt{\frac{3\eta_0}{\phi\rho_0\pi f}}, \quad (S1)$$

where ϕ is the acoustic contrast factor, η_0 and ρ_0 are the dynamic viscosity and density of the surrounding fluid, respectively. f is the driven frequency. The acoustic contrast factor is calculated by:

$$\phi = \frac{5\rho_p - \rho_0}{2\rho_p + \rho_0} - \frac{k_p}{k_0}, \quad (S2)$$

where ρ_p and k_p are the density and compressibility of the particles, respectively, and ρ_0 and k_0 are the density and compressibility of the fluid. Equation S1 is not necessarily entirely prescriptive, however, where the geometry of a system can play a role in the magnitude of the streaming-induced drag force that develops[2,3], the magnitude of this geometry-dependent value is typically on the order of 1. The acoustic radiation force (F_{rad}) dominates when particle size $a > a_c$ [1,4,5] with particles with radius larger than a_c being dominated by F_{rad} and patterned in the acoustic window by the acoustic pressure gradient. Using the parameters for the PDMS particles used here, the a_c is ~ 1 μm, representing the minimum manipulable particle size ($a_{\min}$).

In our device, specific to the case of sound speed measurement, with an effective lateral resolution of 330 μm distributed over a 5 cm transducer aperture, the sound velocity range can be divided into approximately 150 equal parts, which corresponds to a sound velocity change sensitivity of ~5.3 m/s (i.e., 150 divisions of a sound velocity difference of 800 m/s). This sound velocity sensitivity is on the range appropriate to also sense changes in fluid properties including temperature[6] and even pressure [7] that impact sound velocity.

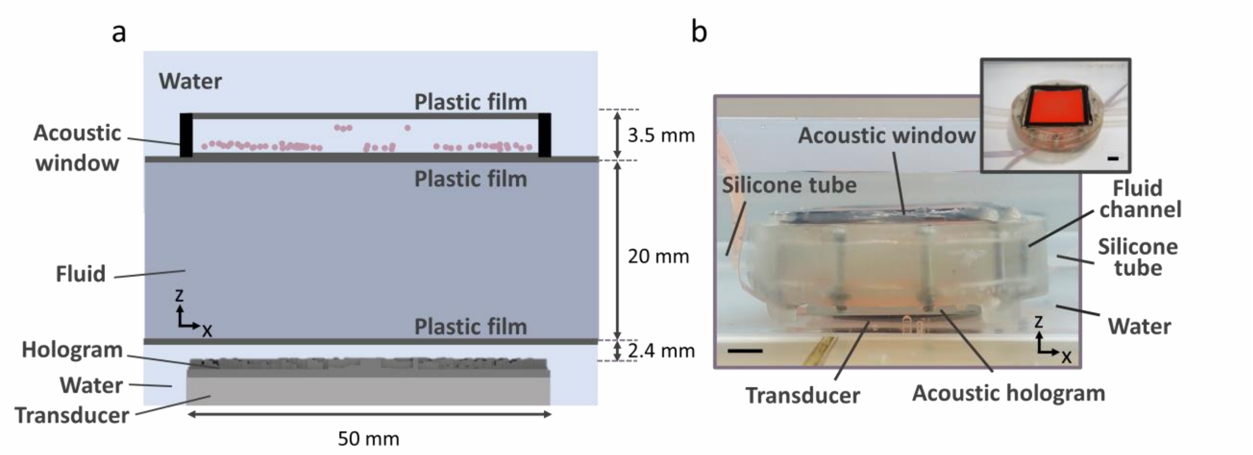

Figure S1. a) Schematic diagram and dimensions of the device. A 2.4 mm height coupling layer filled with water is between the hologram and the fluid channel, where the channel height is 20 mm. A 3.5 mm high acoustic window is sealed at both ends by plastic films and placed on top of the fluid channels. b) Photos of the device. Scale bars are 1 cm.

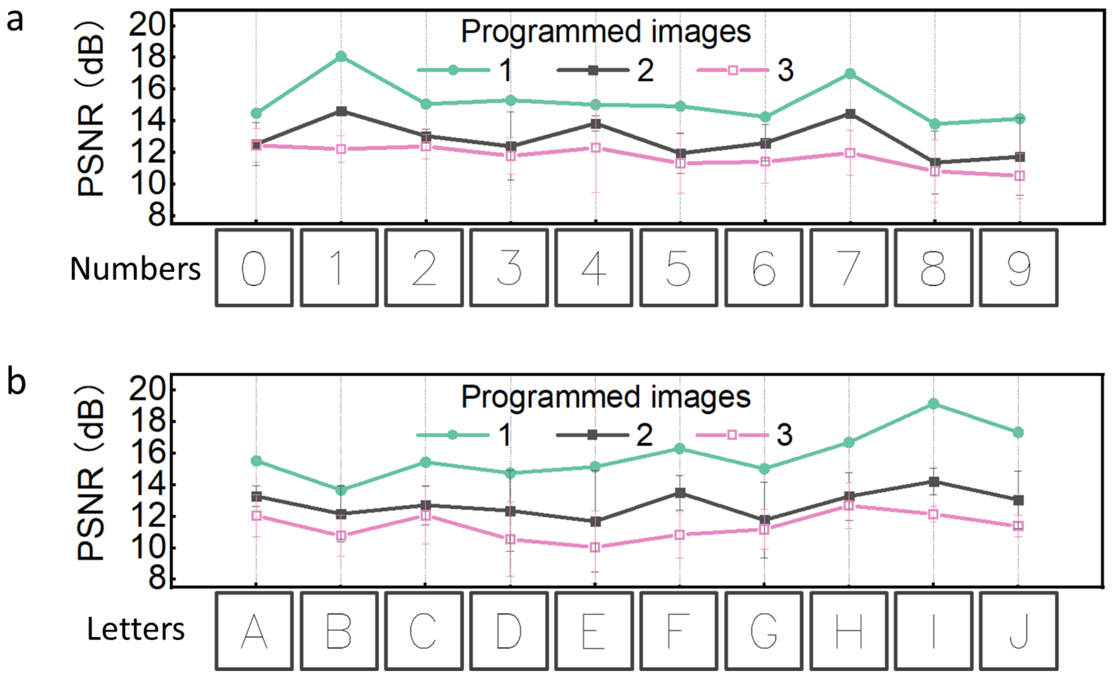

Figure S2. Variation in the implementation of given a) numbers and b) letters and the resulting impact image quality for different numbers of total images encoded in each hologram. The encoded images other than the target image are randomly selected from the listed numbers/letters (excluding the target image).

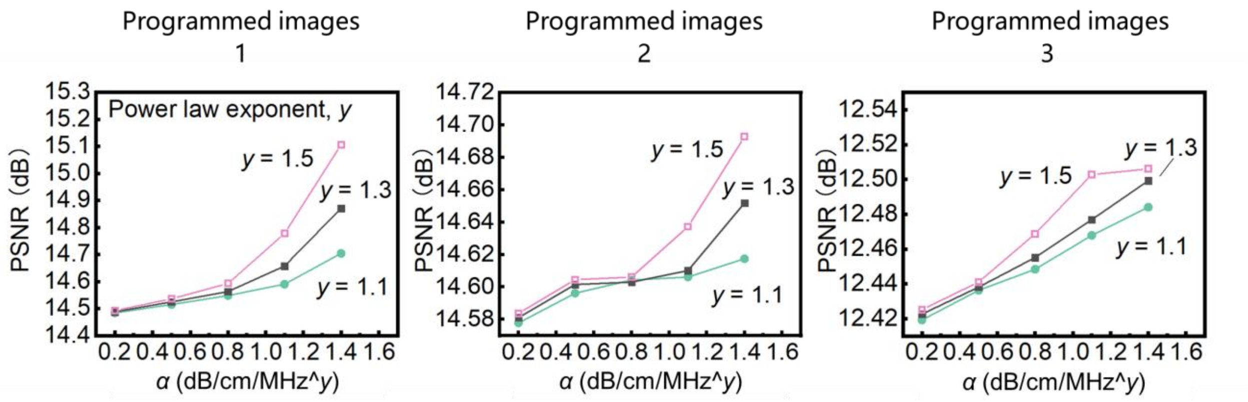

Figure S3. The impact of the attenuation parameters of the medium on image quality, for different power law absorption coefficient (α) and power law exponent (y). The range of α is 0.2-1.6 dB/cm/MHz^y and the range of y is 1.1-1.5.

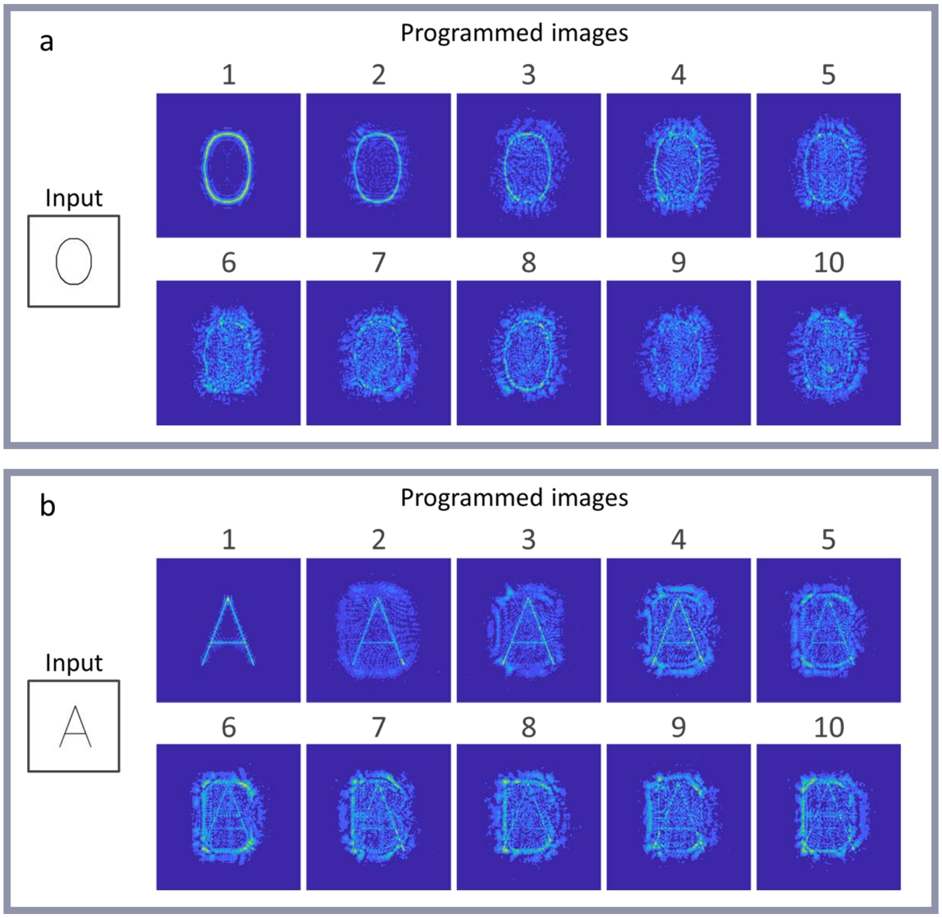

Figure S4. The resulting images in Figure 5b, for a) number "0" and b) letter "A" at the target plane for these images as a function of adding further programmed images.

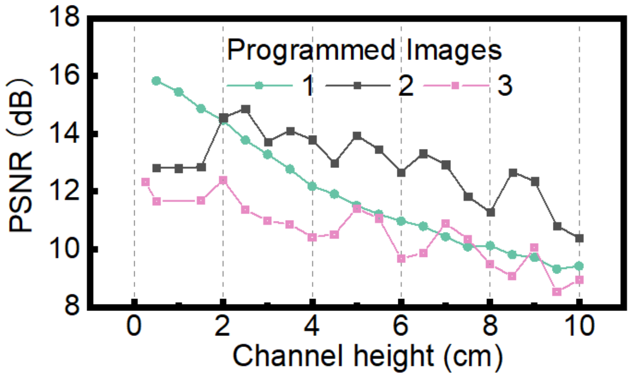

Figure S5. The effect of channel height on image quality, showing that as the channel height increases, the image quality decreases.

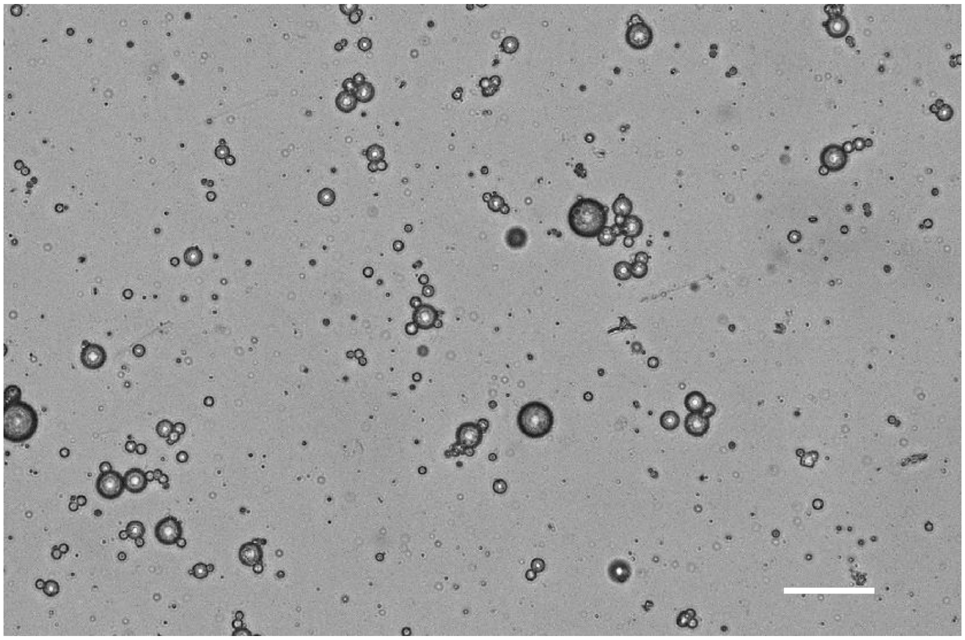

Figure S6. Microscope image of PDMS microparticles with dispersed particle sizes (< 30 μm). Scale bar is 50 μm.

References

[1] F. Barbaresco, L. Racca, L. Spigarelli, M. Cocuzza, S. L. Marasso, C. F. Pirri, G. Canavese, *Nanomaterials* **2021**, *11*, 2630.

[2] D. J. Collins, Z. Ma, Y. Ai, *Anal Chem* **2016**, *88*, 5513.

[3] K. Kolesnik, P. Hashemzadeh, D. Peng, M. E. M. Stamp, W. Tong, V. Rajagopal, M. Miansari, D. J. Collins, *Phys Rev E* **2021**, *104*, 045104.

[4] O. A. Sapozhnikov, S. A. Tsysar, V. A. Khokhlova, W. Kreider, *J Acoust Soc Am* **2015**, *138*, 1515.

[5] P. Zhang, H. Bachman, A. Ozcelik, T. J. Huang, *Annual Review of Analytical Chemistry* **2020**, *13*, 17.

[6] A. H. Smith, A. W. Lawson, *J Chem Phys* **1954**, *22*, 351.

[7] V. A. Belogol’skii, S. S. Sekoyan, L. M. Samorukova, S. R. Stefanov, V. I. Levtsov, *Measurement Techniques* **1999**, *42*, 406.
